# Cell Wall Composition, Biosynthesis and Remodeling during Pollen Tube Growth

**DOI:** 10.3390/plants2010107

**Published:** 2013-03-07

**Authors:** Jean-Claude Mollet, Christelle Leroux, Flavien Dardelle, Arnaud Lehner

**Affiliations:** Laboratoire de Glycobiologie et Matrice Extracellulaire Végétale, EA4358, IRIB, Normandy University, University of Rouen, 76821 Mont Saint-Aignan, France; E-Mails: christelle.leroux@etu.univ-rouen.fr (C.L.); flavien.dardelle@gmail.com (F.D.); arnaud.lehner@univ-rouen.fr (A.L.)

**Keywords:** biosynthesis, callose, cellulose, cell wall, pectin, glycoside hydrolases, pollen tube growth, remodeling, xyloglucan

## Abstract

The pollen tube is a fast tip-growing cell carrying the two sperm cells to the ovule allowing the double fertilization process and seed setting. To succeed in this process, the spatial and temporal controls of pollen tube growth within the female organ are critical. It requires a massive cell wall deposition to promote fast pollen tube elongation and a tight control of the cell wall remodeling to modify the mechanical properties. In addition, during its journey, the pollen tube interacts with the pistil, which plays key roles in pollen tube nutrition, guidance and in the rejection of the self-incompatible pollen. This review focuses on our current knowledge in the biochemistry and localization of the main cell wall polymers including pectin, hemicellulose, cellulose and callose from several pollen tube species. Moreover, based on transcriptomic data and functional genomic studies, the possible enzymes involved in the cell wall remodeling during pollen tube growth and their impact on the cell wall mechanics are also described. Finally, mutant analyses have permitted to gain insight in the function of several genes involved in the pollen tube cell wall biosynthesis and their roles in pollen tube growth are further discussed.

## 1. Introduction

**Figure 1:**
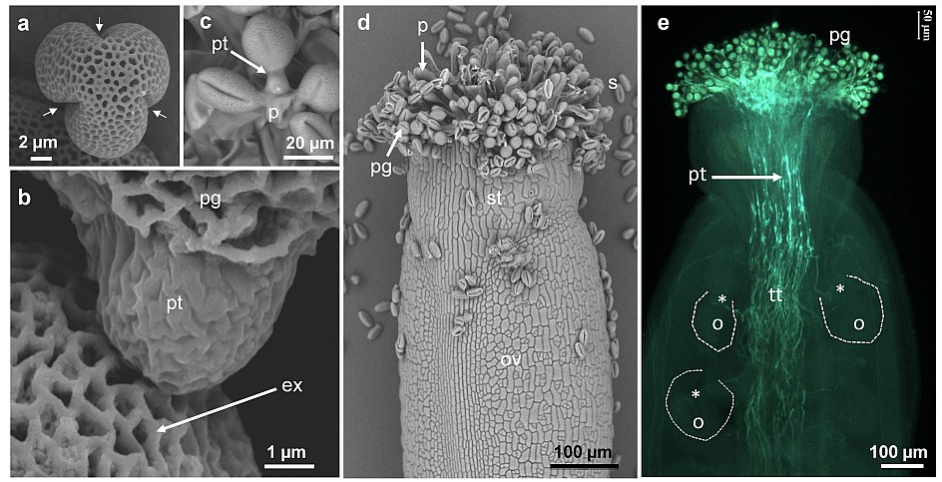
Scanning electron (**a**–**d**) and fluorescent (**e**) micrographs of *Arabidopsis thaliana* (**a**) dry pollen grain showing the three apertures (arrows), (**b**) emerging pollen tube from a pollen grain, (**c**) pollen germination on the papillae, (**d**) self-pollinated pistil with adhering pollen grains on the papillae and (**e**) aniline blue staining of self-pollinated stigma showing pollen tubes within the transmitting tract and reaching the ovules (dashed line and *). ex. exine, p. papillae, o. ovule, ov. ovary, pg. pollen grain, pt. pollen tube, s. stigma, st. style, tt. transmitting tract.

Fertilization of flowering plants requires the delivery of two sperm cells carried by the pollen tube, a fast tip-polarized growing cell, to the egg cell. In plants such as *Arabidopsis thaliana*, this process begins with the adhesion of the pollen grains on the stigmatic papillae after the pollen coat has contacted the papillae (Figure 1a–d). Following hydration of the pollen grain, the pollen tube emerges either from one of the three pollen grain apertures (Figure 1c) or through the exine wall (Figure 1e) [1]. Then, pollen tubes invade the cell wall of the papillae (Figure 1b–d), enter the short style, and grow in the apoplast of the specialized transmitting tract cells (Figure 1b) enriched in extracellular nutrient [2,3].

During this invasive growth, pollen tubes are guided to the ovules via signals that need to pass through the cell wall to reach their membrane-associated or intracellular targets [4,5,6,7,8,9,10,11]. In addition to being the interface between the tube cell and the surrounding (culture medium or female tissues), the cell wall of pollen tubes plays a crucial role in the control of the cell shape, in the protection of the generative cells and in the resistance against turgor pressure induced tensile stress [12,13]. Thus, a tight control of cell wall deposition and remodeling during pollen tube growth is required to fulfill all these functions.

In this review, we describe our current knowledge on the biosynthesis, distribution and biochemistry of cell wall polymers including pectin, hemicellulose, cellulose and callose from several pollen tube species (including plants with dry stigma and solid style like *Arabidopsis thaliana* and tobacco and wet stigma and hollow style like in lily). The structure and functions of arabinogalactan-proteins in pollen tube growth will not be addressed as it was recently detailed by [14]. Finally, the enzymes from the male gametophyte and the female sporophytic counterpart possibly involved in the cell wall remodeling during pollen tube growth are further discussed in relation with the mechanical properties of the cell wall.

## 2. Cell Wall Polymers in Pollen Tubes

Despite the importance of pollen tubes for the delivery of the sperm cells to the egg, little is known about the underlying molecular mechanisms that regulate the mechanical interaction of pollen tubes with the female floral tissues and only very scarce data are available concerning the biosynthesis and remodeling of the pollen tube cell wall.

**Figure 2:**
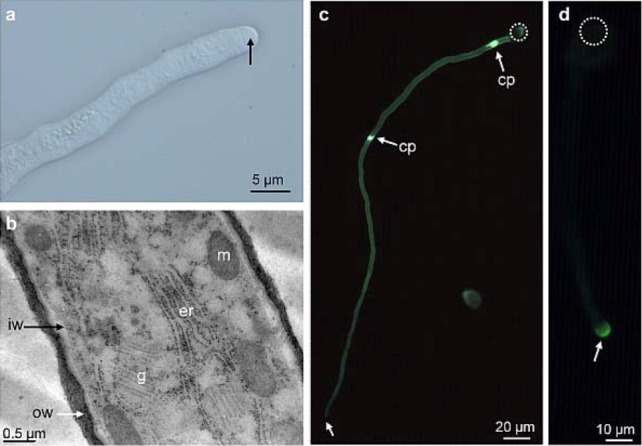
*A. thaliana* pollen tube grown *in vitro*. (**a**) High magnification of eight-hour-old pollen tube grown in liquid medium showing the clear zone at the tip (arrow). (**b**) Transmission electron micrograph of high pressure-freeze substituted pollen tube showing the two cell wall layers. (**c**) Pollen tube stained with decolorized aniline blue showing the callose wall and two callose plugs. Note the absence of staining at the tip (arrow). (**d**) Immunolocalization of highly methylesterified HG with LM20 in a pollen tube showing a strong labeling at the tip (arrow). cp: callose plug, er: endoplasmic reticulum, g: Golgi apparatus, iw: inner wall, m: mitochondria, ow: outer wall. Dotted circle indicates the location of the pollen grain.

Pollen tubes in most species display in the tip region a clear zone like in *A. thaliana* (Figure 2a), composed of numerous Golgi-derived vesicles that migrate toward the apex in the cell cortex and accumulate in an annulus-shaped region adjacent to the extreme tip (apical flank) where they fuse with the plasma membrane to sustain pollen tube growth [15]. At the extreme apex and in the distal region of the pollen tube, endocytosis takes place possibly by clathrin-dependent and -independent pathways [10,16,17,18,19].

Transmission electron microscope observations of *in vitro* and *in vivo* grown pollen tubes from many species including lily [20], tobacco [21,22], *A. thaliana* [23,24,25,26] and in several but not all the gymnosperm investigated species like *Pinus sylvestris* [27], *Podocarpus nagi* or *Pinus banksiana* [28] showed a cell wall composed of two layers at the shank of the pollen tube: a fibrillar outer layer and a weakly electron-dense inner wall (Figure 2b). In contrast, the inner cell wall layer is generally lacking at the pollen tube tip in normal condition [20,23,24,27].

### 2.1. Distribution of Carbohydrate Epitopes in the Pollen Tube Cell Wall

Distribution of pollen tube cell wall polymers was investigated by using mostly cytochemical reagents, enzymes and/or antibodies (Table 1). In most of the immunolocalization studies, monoclonal antibodies (mAbs) are applied on the whole pollen tube [24,25,26,27,28,29,30,31,32], allowing a cell surface labeling that may mislead in the interpretation as epitopes may have been masked by other polymers. To avoid this problem, enzyme treatments were sometimes applied on fixed tubes or pollen tubes were embedded in resin and sectioned [33]. Another possible artifact is caused by the slow penetration of the chemical fixative that arrests pollen tube growth while exocytosis is still ongoing, or the other way round, that arrests of enzymatic reactions in the wall while the tube is still growing.

**Table 1:** List of probes (antibody, cytochemical reagent and enzyme) used to detect pollen tube cell wall polysaccharides.

Probe ^a^	Polysaccharide ^b^	Epitope recognized ^c^	Refs.
**Antibody**			
CCRC-M1	XyG	α-Fuc-(1→2)-β-Gal	[34]
LM15	XyG	XXXG, XXLG, XLXG, XXGG	[35]
JIM5	weakly methylesterified HG	α-MeGalA-(1→4)-α-GalA_(4)_-(1→4)-α-MeGalA	[36]
LM19	weakly methylesterified HG	α-GalA-(1→4)_(4)_	[37]
JIM7	partially methylesterified HG	α-GalA-(1→4)-α-MeGalA_(4)_-(1→4)-α-GalA	[36]
LM20	partially methylesterified HG	α-MeGalA-(1→4)_(4)_	[37]
LM8	xylogalacturonan	unknown	[38]
LM5	(1→4)-β-d-galactan (RG-I)	β-Gal-(1→4)_(3)_	[39]
LM6	(1→5)-α-l-arabinan (RG-I)	Branched α-Ara-(1→5)_(5)_	[40]
LM13	(1→5)-α-l-arabinan (RG-I)	Linear α-Ara-(1→5)_(5)_	[41]
LAMP	callose	β-Glc-(1→3)_(5)_	[42]
Anti-RG-II	Monomeric and dimeric RG-II	unknown	[43]
**Cytochemical reagent**			
Calcofluor white	β-Glucan	na	
Aniline blue	callose	na	[44]
PI	HG	na	[45]
**Protein**			
CBH-I	cellulose	na	[22]
CBM3a	Crystalline cellulose	na	[46]

^a^ CCRC-M. Complex Carbohydrate Research Center-Monoclonal, LM, Leeds Monoclonal, JIM, John Innes Monoclonal, LAMP, LAMinarin Pentaose, RG-II. Rhamnogalacturonan-II, PI. Propidium iodide, CBH-I. Cellobiohydrolase-I, CBM3a. Cellulose binding module3a. ^b^ XyG. Xyloglucan, HG. Homogalacturonan, RG-I. Rhamnogalacturonan-I. ^c^ Ara. arabinose, Fuc. fucose, Gal. galactose, GalA. galacturonic acid, Glc, glucose, MeGalA, 6-*O*-methyl-galacturonate, na. not applicable. For more information see [47].

Distribution of cell wall polymers in pollen tubes was investigated in plant species of the angiosperm eudicots such as *A. thaliana* [23,24,25,26], *Nicotiana tabacum* [21,22,29], *Solanum chacoense* [30], *Camellia japonica* [31], *Torenia fournieri* [32] and *Actinidia deliciosa* [33] as well as angiosperm monocots like *Lilium longiflorum* [48,49] and *Zea mays* [50]. More recent studies have focused on the gymnosperms like *Pinus sylvestris* [27], *Picea meyeri* [51] and several others [28,52].

One of the main differences between the primary cell wall of somatic and pollen tube cells is despite its presence, the low abundance of cellulose, a β-(1→4)-glucan. Instead, callose, a β-(1→3)-glucan, is the main cell wall polysaccharide. Callose is mostly localized in the inner cell wall and is absent from the tube tip of most angiosperm pollen tubes [53,54] including *A. thaliana* (Figure 2c) [25]. In contrast, in *Cycas* and several *Pinus* species, callose was not detected in the pollen tube wall by aniline blue staining [28]. However, in *P. sylvestris*, a strong fluorescence was detected with aniline blue at the tube tip [27]. In addition, callose is deposited at periodic intervals to form callose plugs that maintain the tube cell in the apical expanding region of angiosperm pollen tubes (Figure 2c) and separates the viable from the degenerating region of the tube [12]. In tobacco using CBH-I-gold and LAMP MAb [22] and in *A. thaliana* using CBM3a [26] and LAMP MAb [25], callose and cellulose are localized in the plugs and in the inner cell wall of the pollen tube. In contrast, in long-living and slow-growing gymnosperm pollen tubes, the callose plug deposition is apparently not a consistent feature as well as the permanent callose wall in pollen tubes [52]. This observation suggests that in the evolution of flowering plants a permanent callose wall and callose plug deposition appeared through the short-lived and fast-growing pollen tubes of angiosperms [55]. Cellulose is present in the entire cell wall of pollen tubes with a weaker staining with calcofluor white or CBH-I-gold labeling at the tip in most angiosperms [22,25,27,30]. Recently, Chebli *et al*. [26] using CBM3a that binds to crystalline cellulose found two populations of *A. thaliana* pollen tubes displaying intense or weak labeling at their tips suggesting that the labeling was related to temporal growth rate. Similarly, in *T. fournieri*, the distribution of cellulose changed temporally and spatially, being abundant in freshly germinated pollen tubes, and absent from the tube tip of older pollen tubes [32]. Using atomic force microscopy, Wu *et al.* [32] also revealed that cellulose microfibrils were more abundant in the apical region of slow-growing tubes whereas only few microfibrils were observed in the auxin-stimulated pollen tubes suggesting a correlation between cellulose abundance and pollen tube growth. In Conifers, pollen tubes have cellulose in the entire cell wall and may contain more cellulose deposited at the tube tip, which may explain the slower growth [52,56].

Pectins are complex polymers consisting of homogalacturonan (HG), which can be methyl- and acetyl-esterified, rhamnogalacturonan-I (RG-I), rhamnogalacturonan-II (RG-II), and xylogalacturonan [57]. HG is a polymer of repeated units of (1→4)-α-d-galacturonic acid. Upon block wise action of pectin methylesterases on methylesterified HG, several parallel HG chains can be cross-linked via the interaction of the negative charges of the carboxyl groups of galacturonic acid and calcium [58]. RG-II has a galacturonan backbone with four well defined oligosaccharides composed of unusual sugars such as apiose, aceric acid and 3-deoxy-d-manno-2-octulosonic acid (Kdo) of unknown function [57]. RG-II is present in the primary cell wall of all higher plants and its structure is evolutionarily conserved in the plant kingdom. In the cell wall, it exists predominantly in the form of a dimer that is cross-linked by a borate di-ester between two apiosyl residues [57]. On the other hand, RG-I consists of the repeating disaccharide unit, (1→4)-α-d-galacturonic acid-(1→2)-α-l-rhamnose. On the rhamnosyl residues, a wide variety of side chains can be detected ranging from monomers to large oligosaccharides such as (1→4)-β-d-galactan, (1→5)-α-l-arabinan, and/or type I arabinogalactan [57].

Most of the studies on the pectic wall of the pollen tube focused on the detection of HG domains using the JIM5 and JIM7 mAbs (Table 1). To date, localization data of other pectic domains such as xylogalacturonan and RG-I side chains in the pollen tube wall are scarce. Immunofluorescence labeling of weakly and highly methylesterified HG with JIM5 and JIM7 or the newly introduced LM19 and LM20 depends clearly on the species, the *in vitro* growth condition and the growth rate of the pollen tube [59]. It is however generally observed that in most angiosperms, highly methylesterified HG is mostly detected at the tip (Figure 2d) and weakly methylesterified HG epitopes are preferentially detected either in the entire pollen tube or in the pollen tube except in the tip region as shown in the Brassicacae (*A. thaliana*) [25], Solanaceae (wild potato, tobacco, petunia), Oleaceae (jasmine), Poaceae (corn) [60], Scrophulariaceae (*T. fournieri*) [32] and Liliaceae (lily) [48,49]. Similar observations were obtained in *Picea meyeri* [51] and *Picea wilsonii* [61] pollen tubes. In other gymnosperm species like *P. macrophyllus* and *P. banksiana,* pollen tubes were not labeled with JIM5 and JIM7 using electron microscopy observation [28].

Based on Elisa assays and dot-blots, it was clearly shown that JIM5 and JIM7 as well as LM19 and LM20 have specific but also overlapping binding capabilities to different degrees of methylesterification (DM) of HG domains after random or blockwise action of pectin methylesterases (PME) [37,62]. LM20 binds to HG with DM ranging between 85 and 16%, whereas LM19 binds to HG with DM between 66% and the demethylesterified form [37]. Using these data and assuming that PME has a blockwise action to allow calcium cross-linking in the shank of the tube, we can estimate that the DM of HG at the tip is probably over 70% and back from the tip, the DM is less than 15%. In lily, this change of methylesterification level occurs between 15 and 20 µm from the pole of the pollen tube corresponding exactly to the transition zone between the apical dome and the cylindrical shank of the cell [63]. In *A. thaliana*, this modification takes place closer to the tip (between 3 and 10 µm) [26]. Recently, propidium iodide (PI) was used to probe low methylesterified HG on living *A. thaliana* and *L. longiflorum* pollen tubes, which allowed following the dynamics of the pectin during pollen tube growth. The fluorescence of PI was detected at the tip and the shank of the pollen tubes with PI fluorescence oscillations preceding growth rate oscillations [45]. Finally, in certain species such as lily [49], *Ornithogalum* [59], tobacco [54] and others [60], periodic ring-like labeling patterns of weakly methylesterified HG were clearly visible along the pollen tube and originated from cell wall deposition during the slow growth pulses [54].

Side chains of RG-I are also present in the cell wall of pollen tubes. Using LM6 and/or LM13, epitopes associated with arabinan are evenly distributed along the entire pollen tube in *A. thaliana* [25], *A. deliciosa* [33] and *P. wilsonii* [61]. At the electron microscopy level, arabinan is mostly detected in the outer cell wall layer in *A. thaliana* [25] consistent with the location of other pectic motifs such as HG. Galactan is also detected in *A. thaliana* pollen tubes but the labeling intensity is weak [25] and almost absent in *Picea* [61].

Finally, RG-II, the major boron-binding component of the cell wall, is detected in the entire lily pollen tube cell wall [43] but is not in *Picea* [61].

Xyloglucan (XyG) is the major hemicellulosic polysaccharide of the primary cell wall of angiosperm eudicots and non-commelinid monocots [64,65]. Classic XyG consists of a (1→4)-β-d-glucan backbone substituted with xylose, galactose-xylose or fucose-galactose-xylose motifs [66]. In the primary cell wall, XyG interacts with cellulose via hydrogen bonds and participates in the control of cell expansion [67]. The presence of fucosylated and galactosylated XyG was assessed only recently in *A. thaliana* pollen tubes using CCRC-M1 [25,68] and LM15 [25]. Labeling with both mAbs is detected in the entire pollen tube wall and at the electron microscopy level; it was shown that XyG is present in the outer and inner layers of the pollen tube cell wall [25] suggesting a possible interaction with cellulose microfibrils.

All these data reveal a common organization of the pollen tube cell wall in the species from the angiosperm eudicots and the non-commelinid monocots. In contrast, with the slow-growing and short-traveling gymnosperm pollen tubes, it appears that the distribution of cell wall polymers, especially the callose, is altered [69]. However, more studies using the probes listed in Table 1 are required to draw a general conclusion. To date, the investigation on the distribution of RG-II in pollen tubes is very limited but with the development of new specific probes for each individual side chains, the gap might be soon overcome. Another attracting method that may develop in the near future in the study of cell wall dynamics during pollen tube growth is the use of sugar analogs compatible to click chemistry associated with *in vivo* cell imaging [70]. In this method, sugar analogs are metabolically incorporated into cell wall polymers and subsequently labeled with covalent linkages to fluorescent probes [71] as shown with alkynylated fucose analog incorporated in the pectin of *A. thaliana* root cell wall [72]. This method will allow for insight into the dynamics and recycling of cell wall polymers on living pollen tubes.

### 2.2. Chemical Composition of Pollen Tube Cell Wall

**Figure 3:**
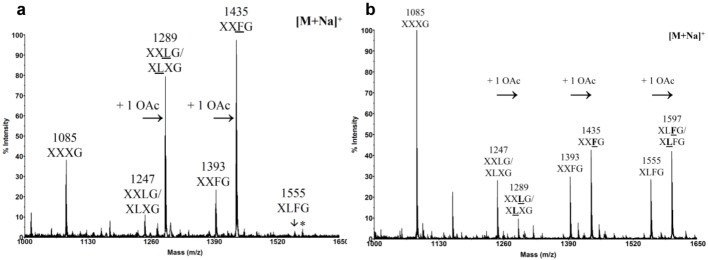
MALDI-TOF mass spectra of *endo*-glucanase-generated XyG fragments from *A. thaliana* (**a**) Spectrum from pollen tubes. (**b**) Spectrum from mature leaves. The structures of the XyG fragments are shown according to the nomenclature proposed by Fry *et al*. [19]. Underlined structures represent *O*-acetylated side chains (+1 *O*Ac). The asterisk indicates the signal of XLFG fragment with K^+^ adduct ion instead of Na^+^. From Dardelle *et al*. [25], Copyright American Society of Plant Biologists [79].

Only very scarce studies have focused on the biochemical characterization of the pollen tube cell wall. The most striking point observed in the few biochemical characterizations of the cell wall of tobacco, *C. japonica*, lily, tulipa and *A. thaliana* pollen tubes is consistently the high level of arabinosyl residues [25,73,74]. In *A. thaliana*, Ara represents 43%, Glc 20%, GalA 11%, Gal 8% and Rha 5% of the total sugars [25]. Linkage analyses show that most of the arabinosyl residues are T-Ara,5-linked and to a lesser extent 2,5-linked. These data indicate that the pectin of *A. thaliana* pollen tube cell wall is composed of 6% HG, 5% RG-I backbone harboring abundant chains of (1→5)-α-l-arabinan. This common feature suggests that arabinan may have an important role during pollen tube growth. Finally, linkage analyses also indicate (1) that most of the Glc is 3-linked indicating the abundance of callose [25,73] and (2) confirm that XyG is also present based on the detection of 4-Glc, 2-Xyl and T-Xyl, T-Gal and Fuc residues [25]. According to the one letter code proposed by Fry *et al*. [75], the unsubtituted glucosyl residue of the backbone is represented by the letter G. X, L and F represent the substitution by xylose, galactose-xylose and fucose-galactose-xylose, respectively. XyG can also be *O*-acetylated generally on the galactose, indicated by an underline letter, but the biological significance of *O*-acetylation is not known yet [76]. Generally, every four or five glucoses of the glucan backbone are not substituted allowing the cleavage of the polymer by *endo*-glucanase. The small oligosaccharides can then be analyzed by MALDI-TOF mass spectrometry, known as OLIMP method (OLIgosaccharide Mass Profiling) [77]. Using this technique, the fine structure of *A. thaliana* pollen tube XyG was recently determined. It revealed significant differences with the XyG from vegetative organs such as leaves (Figure 3). Whereas XXXG is the main fragment of leaf XyG [25,78], the fucosylated and *O*-acetylated fragments (XXFG and XLFG) are the major motifs in *A. thaliana* pollen tube XyG [25] suggesting an important role of these two features in pollen tube growth and/or in the interaction with the female tissues. 

Used in the mid-90s to study the cell wall composition of algae [80] and later widely adopted to screen flax and *A. thaliana* cell wall mutants [81,82], Fourier transform infrared (FT-IR) microspectrometry associated with principal component analysis was recently used to study the effect of hormones, minerals or drugs on the cell wall composition of elongating pollen tubes [51,83]. It shows that treatments of *P. meyeri* pollen grains with brefeldin A, a drug able to inhibit the secretory pathway, reduce the pollen germination and tube growth by disrupting the secretory vesicles at the tip and significantly decrease the content of pectin in the apical region [84]. Treatments with nitric oxide donor or NO synthase inhibitor induce the accumulation of acidic HG and callose in the tip region [85]. Interestingly, blocking the release of intracellular calcium with drugs significantly alters the cell wall structure in *Picea* pollen tubes with the accumulation of callose and the disappearance of methylesterified HG at the tube tip [61]. Finally, treatment of *T. fournieri* pollen tubes with auxin promotes the growth by stimulating the synthesis of pectin, reducing the cellulose density and modifying the orientation of cellulose microfibrils as observed by atomic force microscopy [32]. However, whereas the vibrations associated with cellulose, phenolics, the methylester and the carboxyl groups of GalA are well assigned by FT-IR, the technique does not allow discriminating clearly other pectin motifs such as arabinan and/or galactan side chains of RG-I in the cell wall network. It is, however, a non destructive method that may be convenient to use in the future for determining the overall composition of pollen tube cell wall mutants compared to wild type. It may also be useful to follow the evolution of the cell wall composition in different portions of the pollen tube during growth. 

Due to the technical challenges involved such as the labor intensive work to collect enough material, the number of studies focused on the biochemistry of pollen tube cell wall is limited. As a consequence, it does not permit to assess clearly the differences in the cell wall composition between angiosperm (monocot and eudicot) and gymnosperm. However, with the development of more sensitive equipments such as mass spectrometry, more information may be soon collected.

## 3. Cell Wall Polymer Biosynthesis in Pollen Tubes

### 3.1. Pectin Biosynthesis

Pectin represents one of the main carbohydrate polymers found in the cell wall of pollen tubes. Given the complexity of its structure, it is predicted that 67 different glycosyltransferases, methyltransferases and acetyltransferases are required for its biosynthesis [86]. The synthesis of the different pectin motifs is carried out in the Golgi apparatus and they are secreted via Golgi-derived vesicles [87].

#### 3.1.1. Homogalacturonan

The synthesis of HG requires the activity of GALACTURONOSYLTRANSFERASEs (GAUTs) that was biochemically demonstrated in *Petunia axillaris* pollen tubes [88]. In *A. thaliana*, the multigenic family of *GALACTURONOSYLTRANSFERASE (GAUTs)* is composed of 15 genes [86,89] whose members are related to another family representing the *GALACTURONOSYLTRANSFERASE-LIKE* (*GATLs*), composed of 10 genes [90]. *GAUT1* (CAZy family GT8) is a galacturonosyltransferase gene that first was shown to be functionally implicated in the HG synthesis in *A. thaliana* vegetative organs [91]. The inactivation of *GAUT8* induces a reduction of the level of HG epitopes, a decrease of the amount of GalA in the cell wall of vegetative organs and a partial sterile phenotype [92]. Among all these genes, 13 *GAUTs* and 8 *GATLs* are expressed in the inflorescence of *A. thaliana* [89,90]. According to transcriptomic data, only two *GAUTs* (*GAUT13* and *GAUT14*) and two *GATLs* (*GATL4* and *GATL7*) are expressed in pollen tubes [90,93]. Interestingly, the *GALT4* expression seems to be pollen specific and is highly expressed in *A. thaliana* pollen tubes [90], which may suggest its implication in the synthesis of HG in pollen tubes but functional studies are necessary to assess the role of these genes in pollen tube growth.

#### 3.1.2. Xylogalacturonan

In *A. thaliana*, the gene At5g33290 is involved in the xylogalacturonan biosynthesis, but the *xgd1* (*xylogalacturonan deficient1*) mutants do not display any visible pollen or pollen tube phenotype [94]. Other xylogalacturonan biosynthesis genes are undoubtedly implicated in this process but it remains to be established.

#### 3.1.3. Rhamnogalacturonan-I

The backbone of RG-I is made of a repeating disaccharide α-(1→4)-d-GalA-α-(1→2)-l-Rha with Rha residues that can be substituted with galactan, arabinan and/or arabinogalactan [95]. In the arabinan side chain, arabinosyl residues are almost entirely in the *furanose* configuration. Interestingly, rice REVERSIBLY GLYCOSYLATED PEPTIDEs (RGPs) show strong amino acid sequence identity (~80%) with an UDP-arabino*pyranose* mutase implicated in the interconversion of UDP-Ara*p* and UDP-Ara*f* [96]. Moreover, in *A. thaliana*, double knock-out mutations in two *RGPs* (*RGP1* and *RGP2*) showed a strong defect in the inner pollen wall and pollen lethality [97] suggesting that *RGP1* and *RGP2* act redundantly during pollen development. More recently, Rautengarten *et al.* [98] have shown that RGP1 and RGP2 are cytosolic and able to perform this interconversion indicating that RGPs may be part of the arabinan biosynthesis network. However, further studies are required to verify if RGP1 and RGP2 are also involved in the growth of pollen tubes. Moreover, biochemical evidence is needed to verify if the arabinan side chains of RG-I in the mutants are structurally different to the one found in wild type pollens and if other cell wall molecules containing Ara residues such as arabinogalactan proteins are not also affected.

#### 3.1.4. Rhamnogalacturonan-II

Studies of the possible involvement of RG-II in pollen tube growth are very recent. In 2006, it was shown in tobacco that the mutation in *NpGUT1* (*Nicotiana plumbaginifolia GLUCURONOSYLTRANSFERASE1*) resulted in the inhibition of pollen tube elongation, presumably due to an abnormal deposition of RG-II and boron in the pollen tube tip cell wall [99]. However, it was shown recently that in *A. thaliana*, IRX10 (IRREGULAR XYLEM10) and IRX10-L (IRREGULAR XYLEM10-LIKE) proteins, which are closely related to *Np*GUT1, play a critical role in the synthesis of glucuronoxylan and not RG-II. [100,101]. To our knowledge, no report has shown any glucuronoxylan in the cell wall of *A. thaliana* or tobacco pollen tubes and these data contrast with the proposed function of *Np*GUT1 in the RG-II synthesis in pollen tubes.

Recently, three other genes involved in the biosynthesis of RG-II were shown to play an important role during *A. thaliana* pollen tube elongation. *KDO-8-P* *SYNTHASEs* (*AtKDSA1* and *AtKDSA2*) are involved in the synthesis of Kdo, one of the rare sugars composing the RG-II, with eight carbons. *Atkdsa1* and *Atkdsa2* double pollen mutants in *quartet* background are unable to form an elongated pollen tube properly and to perform fertilization [102]. In 2010, two *male gametophyte defective* (*mgp*) mutants, impaired in the biosynthesis of RG-II were isolated: *mgp4*, knock-out for a RG-II XYLOSYLTRANSFERASE [103] and *mgp2*, knock-out for a SIALYLTRANSFERASE-LIKE [104]. Both mutants display similar phenotypes with a strong inhibition of pollen germination and a delayed pollen tube growth *in vitro* and *in vivo* compared to wild type. Because the three *A. thaliana* sialyltransferase-like proteins, homologous to mammalian sialyltransferases, do not show any sialyltransferase activity *in vitro* and that the nucleotide sugar donor of the sialyltransferase CMP-Sia is similar to CMP-Kdo, Deng *et al*. [104] suggested that these genes may be involved in the RG-II biosynthesis pathway. Another contribution pointing out the critical role of RG-II in pollen tube growth was recently brought by Kobayashi *et al*. [105]. They studied the gene coding for the enzyme CTP:Kdo cytidylyltransferase (CMP:Kdo Synthetase, CKS) that activates Kdo as a nucleotide sugar during the biosynthesis of RG-II. They showed that the protein is located in the mitochondria and that this gene is essential for pollen tube development as the mutation in *CKS* induces the inhibition of pollen tube elongation [105]. All these studies indicate that RG-II is important for pollen tube growth but none of the studies have shown biochemically that the RG-II structure in the pollen or the pollen tube was impaired. The development of specific probes targeting the different side chains of RG-II should facilitate studies on this non-abundant but important pectin motif in pollen tube growth but to date such tools are not available.

#### 3.1.5. Pectin Methyltransferase and Pectin/XyG Acetyltransferase

The transferase activity of methyl and acetyl groups on HG was shown *in vitro* [106,107]. *QUASIMODO2* and *3* (*QUA2* and *QUA3*) are two genes coding for putative HG methyltransferases but their biochemical activities and their function during pollen tube growth have not been confirmed [108,109]. In 2011, a mutant designated as *reduced wall acetylation2* (*rwa2*) was studied. The loss-of-function in the *RWA2* gene was accompanied by a decrease of the level of acetylated cell wall polymers [110] but no phenotype was observed on the pollen tube indicating that other genes are likely to be involved in this process.

### 3.2. Xyloglucan Biosynthesis

Based on the structure of XyG, its biosynthesis requires a combination of (1→4)-β-glucan synthases, (1→6)-α-xylosyltransferases, (1→2)-β-galactosyltransferases, (1→2)-α-fucosyltransferases and *O*-acetyltransferases [111]. In *A. thaliana*, *CSL4* codes for a (1→4)-β-glucan synthase involved in the synthesis of the XyG backbone but transcriptomic data indicate that this gene is not expressed in pollen [112]. At least five *XYLOGLUCAN XYLOSYLTRANSFERASEs* (*XXTs*) have been identified in *A. thaliana* [76,113,114] but their implications during pollen tube growth have not been assessed yet. Two *XYLOGLUCAN GALACTOSYLTRANSFERASEs* were also characterized: *MUR3* in *A. thaliana* [115] and more recently *XLT2* in *Tropaeolum majus* [116]. However, none of these genes is expressed in pollen grains or pollen tubes [93]. *MUR1* codes for an enzyme that catalyzes the first step of the biosynthesis of GDP-fucose and the *mur1* mutants show a lack of labeling of fucosylated XyG epitopes with CCRC-M1 in hypocotyls, shoots or leaves [68]. However, the mutation does not affect the distribution of the fucosylated XyG epitopes in the pollen tube suggesting that this gene is not implicated in the XyG biosynthesis in pollen tubes. Mutation in the *FUCOSYLTRANSFERASE* gene (*FUT1*, *FT1* or *MUR2*) eliminates the fucosyl residues on the XyG side chains in all major plant organs indicating that FUT1 is involved in most of the XyG fucosyltransferase activity in *A. thaliana* [117,118]. *MUR1* and *FUT1* are not expressed in pollen grains or pollen tubes but are strongly expressed in the stigma and in ovarian tissues in *A. thaliana* [93] suggesting that XyG from the female tissues may be highly fucosylated but more direct experimental evidence is required.

### 3.3. Cellulose Biosynthesis

It is generally assumed that cellulose microfibrils are assembled by cellulose synthases (CESs) located at the plasma membrane in a form of rosette terminal complex. However, the possibility that the first step of cellulose biosynthesis begins in the Golgi apparatus in higher plants cannot be ruled out [119]. Different multigenic families are implicated in cellulose biosynthesis. Ten CESA (CELLULOSE SYNTHASE A) genes were found in the A. thaliana genome and it was shown by mutant analyses that they play distinct roles in the cellulose synthesis process [120,121]. Another multigenic family, CSLD (CELLULOSE SYNTHASE LIKE-D), related to CESA, seems also to be involved in cellulose biosynthesis and contains six members in the A. thaliana genome [122,123]. In rice, the genome contains 7 CESA and 5 CSLD genes [124]. In tobacco, NaCSLD1 (Nicotiana alata *CELLULOSE SYNTHASE-LIKE D1*) was found to be only expressed in the anther and *in vitro*-grown pollen tubes and was predicted to code for a cellulose synthase in pollen [125]. In *N. tabacum* pollen tubes, CESA and CSLD were detected along the entire length of the pollen tubes with a higher concentration at the apex [126]. Similarly, in A. thaliana, CESA6, CSLD1 and CSLD4 were found at the plasma membrane of the pollen tube both at the tip and in the shank [26,127]. Moreover, crystalline cellulose was also found inside cytoplasmic vesicles and the trans Golgi network in A. thaliana pollen tubes leading to the hypothesis that the synthesis of short cellulose microfibrils may initiate in this compartment giving the pollen tubes the head start in assembling the cell wall necessary to promote rapid elongation [26]. Alternatively, the vesicles containing the cellulose may originate from endocytosis suggesting that cellulose in the shank of the tube may be degraded and recycled [26].

The CESA1 and CESA3 mutations are both gametophytic lethal. 50% of pollen grains from the heterozygous cesa1+/− or cesa3+/− plants are significantly deformed and do not produce pollen tube [128]. The cesa6 null mutants display subtle growth defect and the pollen is not deformed [128]. On the other hand, the triple cesa2/6/9 mutants are sterile and pollen grains are strongly deformed [128]. The data indicates that *CESA2* is functionally redundant with *CESA6*. As CESA9 is strongly expressed in pollen grains, these studies suggest that *CESA1*, *3*, *6* and *9* play a critical role during pollen grain formation. Recently, it was shown using pCESA6::GFP-CESA6 construction that CESA6 is expressed in pollen tubes indicating that in addition to CESA1, 3 and 9, CESA6 may also play a role during pollen tube growth [26].

Three genes from the CSLD family are also implicated in pollen tube growth. *Csld1* and *csld4* homozygous mutants are sterile. Mutant pollen grains show abnormal germination and reduce pollen tube growth *in vitro* and *in vivo*. In addition, high level of pollen tubes is bursting due to a reduced cellulose content, abnormal callose deposition and thickened and highly irregular cell wall [123,127]. Finally, mutation in CSLC6 results in a strong reduction of pollen tube growth [129]. All these data support the fact that despite the low abundance of cellulose in the cell wall of pollen tubes, it has an important function in maintaining the integrity of the tube cell.

### 3.4. Callose Biosynthesis

Callose is also synthesized at the plasma membrane by callose synthases (CALSs) located at the apex and the distal regions of tobacco pollen tubes. In longer pollen tubes, CALS accumulates also close to the callose plug [126]. Twelve putative *CALS* genes (*CALS1-12*) have been identified in the *A. thaliana* genome [130]. Among these 12 genes, *CALS5* is implicated in the normal deposition and patterning of the exine pollen grain [131,132] but opposite data are obtained concerning the pollen viability and the ability of the pollen tube to grow normally. *Na*GSL1 (*Nicotiana alata* GLUCAN SYNTHASE-LIKE1) was shown to be a callose synthase [133] involved in the control of callose synthesis during pollen tube growth [134]. In 2011, a study brought evidence that *CALS5* orthologues are expressed in pollen grains of many different angiosperms and gymnosperms but *CALS5* was only expressed in fast-growing pollen tubes (*i.e.*, angiosperms), suggesting that CALS5 plays different but crucial roles during pollen formation and/or germination and pollen tube growth [55]. This study also supports the reports showing that the callose wall in slow-growing pollen tubes is generally transiently detected and the callose plug has so far not been observed in gymnosperm pollen tubes. Based on such variability and transient expression of callose in the pollen of *Pinus*, Pacini *et al*. [135] suggested that the callose may not have a structural function but rather serves as a reserve polysaccharide. In contrast, in fast growing pollen tubes like in *S. chacoense* and *Lilium orientalis*, callose was shown to have an important mechanical function [53].

## 4. Cell Wall Remodeling during Pollen Tube Growth

### 4.1. Cell Expansion: Xyloglucan-Cellulose Reassembly

#### 4.1.1. Expansins

Expansins were first discovered in cucumber hypocotyls [136] and oat coleoptiles [137]. According to Sampedro and Cosgrove [138], expansins belong to a superfamily composed of four families: (1) EXPANSIN A (EXPA), also called α-expansin; (2) EXPANSIN B (EXPB), also known as β-expansin; (3) EXPANSIN-LIKE (EXLA); and (4) EXPANSIN-LIKE B (EXLB). α- and β-expansins are involved in cell expansion: EXPAs may promote separation of cellulose microfibrils by inducing local dissociation and slippage of XyG on the surface of the cellulose, whereas EXPBs work on different polymers, maybe xylan, with a similar effect [139]. The precise role of EXLA and EXLB has not been has not been established yet [138] and to date, no enzymatic activity has been detected for these proteins [140,141].

The *A. thaliana* genome contains 36 genes encoding putative EXPs [141,142]. Expression profile analyses of the transcripts reveal that two expansin genes one α-(*EXPA4*) and one β-(*EXPB5*) are strongly expressed in dry pollen grains, during pollen imbibition and pollen tube growth (Table 2). In addition, four other *EXPs* are strongly expressed in the stigma and one in the ovary of *A. thaliana* (Table 2). To date and to our knowledge, no study has pointed out the role of expansins in the remodeling of the pollen tube cell wall in eudicot plants. Most of the work on expansins comes from the Poales in which the development of pollen grains is accompanied by the expression of *EXPB*. In rice, several putative EXPBs were found in pollen grains by proteomics approach [143] and 4 isoforms were purified from the maize pollen [144]. During the maturation of wheat and triticale male gametophytes, a strong expression of two EXPB genes is detected but unlike the β-expansins from maize, they are not expressed in the mature pollen [145,146]. Even if the exact function of pollen EXPBs remains unanswered, Cosgrove *et al*. [147] suggested that they are released from the pollen grain on the pistil to soften the cell wall of the stigma thus facilitating the penetration and the growth of the pollen tube. Some others hypothesized that EXPBs are implicated in the formation of the exine wall during the male gametophyte formation [145].

**Table 2:** Expression of expansin and xyloglucan *endo*-transglucosylase hydrolase genes in pollen grains, pollen tubes and the pistil of *A. thaliana*. Data were collected from eFP Browser [148]. Proteins are named according to Magrane and the UniProt consortium [149] and according to Hende *et al*. [150] for the expansin family. Pollen grain and pollen tube data are from Qin *et al*. [93] and pistil data from Swanson *et al*. [151]. When the level of expression was <50, the data are not shown.

Cell wall metabolism	Locus	Protein name	Expression level					
			Pollen grain		Pollen tube		Pistil	
			Dry	Imbibed	4 h *in vitro*	Semi *in vivo*	stigma	ovary
**Cell expansion**								
CBMs	At1g20190	Expansin A11 (EXPA11)	<50	<50	<50	<50	1,614.7	421.6
	At1g26770	Expansin A10 (EXPA10)	<50	<50	<50	<50	1,893.9	641.8
	At2g28950	Expansin A6 (EXPA6)	<50	<50	<50	<50	1,919.8	1,614.2
	At2g39700	Expansin A4 (EXPA4)	4,069.6	3,962.1	3,950.8	2,106.8	321.3	429.6
	At3g45970	Expansin-like A1 (EXLA1)	<50	<50	<50	<50	1,462.7	322.1
	At3g60570	Expansin B5 (EXPB5)	1,020.2	1,139.5	1,347.1	3,002.9	53.9	52.7
**Hemicellulose reassembly**								
GH16	At2g06850	XTH4	<50	<50	<50	<50	129.8	1,174.4
	At4g03210	XTH9	<50	<50	<50	<50	785.1	2,518.8
	At4g30270	XTH24	<50	<50	<50	<50	146.5	1,975.7
	At5g65730	XTH6	<50	<50	<50	<50	752.1	1,013.4
	At1g32170	XTH30	1,286.7	346.2	354.2	378.6	167.2	86.2
	At4g18990	XTH29	1,02.2	95.8	91.7	<50	<50	<50

CBMs. Carbohydrate-Binding Modules, GH. Glycoside hydrolase, XTH. Xyloglucan *endo*-transglucosylase hydrolase. 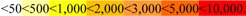

#### 4.1.2. Xyloglucan *endo*-Transglucosylase Hydrolase

The two known activities of XyG *endo*-transglucosylase hydrolase proteins (XTHs) are XyG *endo*-transglucosylase (XET) and XyG *endo*-hydrolase (XEH) [152]. The XET activity consists of cleaving XyG polymers and joining the newly generated end to another XyG chain whereas XEH activity hydrolyzed XyG polymers [152]. XET activity was detected in extracts of the growing portions of various eudicots (including pea, lupin, tomato, sycamore, cow parsley, dandelion and bean), monocots (chive, maize, brome grass and Yorkshire fog) and Bryophytes (liverwort and moss) [153,154]. XTHs are believed to play a central role in the construction and the disassembly of the cell wall architecture [152]. XTHs are encoded by large gene families in land plants and *A. thaliana* and rice contain 33 and 29 *XTH* genes dispersed across their genomes, respectively [155,156]. Among the 33 *XTHs* in the *A. thaliana* genome, 4 are strongly expressed in flowers. Becnel *et al.* [157] have studied the expression of the *XTH* genes using *XTH::GUS* constructions and they showed that *pXTH29::GUS* and *pXTH30::GUS* activities are clearly detected during the anther development and in the mature pollen grain [157]. In contrast, *pXTH1::GUS* and *pXTH33::GUS* activities are restricted to very young flowers. Microarray data extracted from EFP browser are consistent with these results (Table 2). Moreover, expression profiles of *XTH29* and *XTH30* appear to be pollen specific suggesting a specialized function during pollen development.

However, to date, no evidence demonstrates a role of XTHs in the pollen tube growth and cell wall remodeling. Only one *XTH* was linked to pollination. The loss of function of *AtXTH28* led to a dramatic decrease in seed setting due to the inability of the plants to self-pollinate. This phenotype was caused by a net reduction of the stamen filament length due presumably to a reduced capability of the filament cells to expand [158].

### 4.2. Pectin Methylesterase and Pectin/Xyloglucan Acetylesterase

HG is synthesized in the Golgi apparatus and deposited in the expanding tube tip in a highly methylesterified form [159]. The methylesterified HG in the apical zone is thought to provide sufficient plasticity for sustaining the pollen tube growth [15]. Upon block-wise action of PMEs, de-methylesterified HG polymers can form multimers via ionic bonds between the negatively charged carboxyl groups of several HG and Ca^2+^ ions forming a pectate gel that may provide rigidification of the pollen tube cell wall [12,58]. Therefore, the control of the cell wall plasticity by PMEs is critical to ensure a proper fertilization. Conversely, upon random action of PMEs, the partial removal of methylester groups may allow the pectin-degrading enzymes, polygalacturonases (PGs) or pectate lyases (PLs) (see lower section) to cleave the HG backbone thus affecting the rigidity of the cell wall [58,160]. This sequential action of PME and PG was demonstrated in the *quartet* mutants that release pollen grains as tetrads due to the persistence of pectic polysaccharides [161]. The *QUARTET1* gene encodes a PME that is required in association with the *QUARTET3* encoding a PG for the degradation of the HG of the tetrad resulting in the normal pollen separation during microsporogenesis [162,163].

The *A. thaliana* genome contains 66 putative *PMEs*. Most of the genes encoding PMEs display a tissue-specific expression pattern, especially for 14 of them that are specifically expressed in pollen grains or pollen tubes (Table 3) [93,164]. Moreover, proteomics studies of germinated pollen grains from maize, wheat, rice and *A. thaliana* have identified two putative PMEs in germinated maize pollen [165], one in rice [143] and at least one in *A. thaliana* pollen tubes [166]. In tobacco pollen tubes, biochemical analyses have shown the presence of seven isoforms with a wide isoelectric point range [167]. Two PME isoforms were co-localized with methylesterified HG in the cell wall and Golgi-derived vesicles suggesting that PMEs are transported to the tip under an inactive precursor form [167].

The first functional characterization of *PME* genes and their critical roles in pollen tube growth were obtained from two knock-out mutants *vanguard1(vgd1)* [168] and *atppme1* [169]. The disruption of *VGD1* resulted in the burst of pollen tubes *in vitro* and marked retardation of the *vgd1* pollen tube elongation in the pistil resulting in a strong reduction of male fertility and seed set [168]. The lack of AtPPME1 activity in knock-out mutants affects the shape and the growth rates of the pollen tubes, indicating that AtPPME1 is required for the integrity of the cell wall and for the tip-polarized growth [169]. Moreover, when PME from orange peel is exogenously applied to pollen tubes of *Lilium formosanum* and tobacco or when PME is overexpressed, the pollen tube growth is inhibited [170].

**Table 3:** Analyses of the expression profile of pectin methylesterase and pectin and/or xyloglucan acetylesterase genes in pollen grains, pollen tubes and the pistil of *A. thaliana*. Data were collected from eFP Browser [148]. Proteins are named according to Magrane and the UniProt consortium [149]. Pollen grain and pollen tube data are from Qin *et al*. [93] and pistil data from Swanson *et al*. [151]. If the level of expression was <50 for all the selected tissues, data are not shown.

Cell wall metabolism	Locus	Protein name	Expression level					
			Pollen grain		Pollen tube		Pistil	
			Dry	Imbibed	4 h *in vitro*	Semi *in vivo*	stigma	ovary
**Esterase**								
CE8	At1g69940	PPME1	7,600.7	5,098.6	4,702.65	3,209.4	<50	<50
	At2g26450	PME13	4,168.8	4,391.9	4,361.9	4,954.5	<50	<50
	At2g47030	PME4/VGDH1	9,592.1	7,074.2	6,539.6	7,813.1	164.1	<50
	At2g47040	PME5/VGD1	9,592.2	7,074.3	6,539.6	7,813.1	164.1	<50
	At3g05610	PME21	7,557.3	4,778.3	4,618.9	5,347.8	<50	<50
	At3g06830	PME23	4,485.1	1,952.0	1,593.5	73.6	<50	<50
	At3g14310	PME3	<50	<50	<50	<50	284.2	1,047.5
	At3g17060	PME67	4,796.4	4,707.4	4,826.6	4,838.3	<50	<50
	At3g49220	PME34	<50	<50	<50	<50	1,370.3	1,408.2
	At3g62170	PME37	6,829.9	5,434.6	5,566.4	868.0	<50	<50
	At4g15980	PME43	1,930.8	905.9	592.5	<50	<50	51.5
	At4g33230	PME45	759.8	904.1	917.7	92.9	<50	<50
	At5g07410	PME48	7,600.8	5,098.7	4,702.65	3,209.4	<50	<50
	At5g07420	PME49	3,519.5	3,605.9	3,144.6	132.4	<50	<50
	At5g07430	PME50	8,606.5	7,181.6	7,258.6	7,198.7	90.3	<50
	At5g27870	PME28	2,859.6	2,817.7	2,537.8	66.6	<50	<50
	At5g47500	PME68	<50	<50	<50	<50	687.9	1,308.9
	At5g49180	PME58	<50	<50	<50	1,361.1	446.2	1,175.3
CE12	At4g19410	Putative PAE	<50	<50	<50	<50	438.9	1,095.7

CE. Carbohydrate Esterases, PAE. Pectin AcetylEsterase, PME. Pectin MethylEsterase, PPME. Pollen Pectin MethylEsterase, VGD. Vanguard, VGDH. Vanguard Homolog. 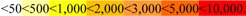

The main regulators of PMEs are PME inhibitors (PMEIs). Sequence analyses indicate that several PMEs contain, in addition to the catalytic domain, an *N*-terminal extension domain (the PRO region) showing similarity with PMEI domains [58]. Based on this, PMEs are classified in two distinct groups depending on the presence or absence of this putative PMEI domain. Group 1 PMEs do not have the PRO region, whereas PMEs from group 2 can contain from 1 to 3 PMEI domains [169]. It is hypothesized that the PRO region is cleaved during the maturation of PME, as so far, only PMEs lacking this domain were found in the cell wall [58].

Recently, Wolf *et al.* [164] indicated that the PRO region of the group 2 PMEs might regulate the release of the mature PMEs from the Golgi apparatus. It was also suggested that the PRO region might play an auto-inhibitory role during maturation [170]. PMEIs are not only found in the PRO region of PMEs but also exist as individual proteins sharing strong sequence similarities with invertase inhibitors (Inv inhibitors). However, there is no clear evidence of a similar function and mode of action between PMEIs/Inv inhibitors and PMEI domains of PMEs. The *A. thaliana* genome contains 76 genes coding highly putative PMEIs/Inv inhibitors. The expression patterns of *PMEIs/Inv inhibitors* are all regulated in a tissue-specific manner [171,172] and transcriptomic data reveal that *PMEIs/Inv inhibitors* are highly expressed in the *A. thaliana* pollen compared to other tissues [171,173]. As 9 out of the 14 *PMEs* specifically expressed in pollens are from the group 2 (with PMEI domains), it may suggest that PMEIs play a crucial role in regulating the activity of PMEs during pollen germination and pollen tube growth. Suppression of the expression of At1g10770 (coding for a putative PMEI) leads to a partial male sterility reducing the seed set by inhibition of pollen tube growth [174]. Moreover, treatments of pollen tubes with exogeneous PMEI also result in an abnormal germination and burst of pollen tubes in *A. thaliana* [175]. Finally, it was shown that AtPPME1 and AtPMEI2, both pollen specific, physically interact and *in vitro* assays revealed that AtPMEI2 was able to inactivate AtPPME1 [176]. The transient expression of AtPPME1 and AtPMEI2 in tobacco pollen tubes demonstrated that AtPPME1 accumulates uniformly from the tip to the shank of the pollen tube. In contrast, the localization of AtPMEI2 is restricted to the tip and in endosomal vesicles suggesting its internalization and recycling [176]. PMEs are thought to play an important role during the tip-polarized growth of the pollen tube by controlling the mechanistic properties of the cell wall. However, the function of PMEs is probably not restricted to the change of the mechanical properties of the cell wall. Weakly methylesterified HG have been implicated in other important physiological processes such as cell attachment in vegetative cells or organs [92,177,178] and in lily, associated with a stigma/style cysteine-rich adhesin (SCA), a secreted plant lipid transfer protein (LTP), in pollen tube adhesion [179,180]. Several *LTPs* are expressed in the transmitting tract of the *A. thaliana* pistil along the pollen tube path [181] that may be involved in this process. In tobacco, it was shown that TobLTP2 purified from the stigma exudates accumulate in the pistil and are able, as expansins do, to promote cell wall loosening. However, the pollen tube growth in LTP-silenced plants is similar to wild type plants, suggesting either that pollen tubes do not require this loosening protein or that other loosening agents may have act redundantly [182]. Recently, LTP5, produced in *A. thaliana* pollen tubes and in the transmitting tract, was proposed to play important roles in maintaining the cell polarity at the tube tip and adhesion-mediated guidance perhaps by interactions with pectins [183]. Direct evidence is lacking, but it appears highly probable that PMEs, by modifying the degree of methylesterification of HG, play a direct role in adhesion and guidance of the pollen tube.

Finally, one putative pectin acetylesterase was detected in rice pollen [143] and a recent report has shown that the overexpression of the *Populus PECTIN-ACETYLESTERASE1* in tobacco affected the shape of the pollen grains and their abilities to germinate [184].

### 4.3. Pectin lyase

*PL (PECTIN LYASE) and PLL (PECTIN LYASE-LIKE)* genes are also abundantly expressed in tomato, tobacco, alfalfa and arabidopsis pollen [185,186,187] and several pollen allergens have PLL activities [188]. Out of the 26 *PLL* genes, 14 are expressed in pollen [189]. None of these 14 genes is specifically dedicated to the male gametophyte but 4 of them are strongly expressed in pollen grains (Table 4). To our knowledge, there is no experimental evidence of the implication of *PLL* in the remodeling of the pollen tube wall. Many promoter activities of the *PLLs* genes are similar to those exhibited by many *POLYGALACTURONASEs* (*PGs*) [190]. This may imply a close functional association between PLLs and PGs, particularly in the digestion of the pollen grain cell wall prior to germination and during pollen tube growth [190,191].

**Table 4:** Analyses of the expression profile of *PECTATE LYASEs* in pollen grains, pollen tubes and in the pistil of *A. thaliana*. Data were collected from eFP Browser [148]. Proteins are named according to Magrane and the UniProt consortium [149]. Pollen grain and pollen tube data are from Qin *et al*. [93] and the pistil data from Swanson *et al*. [151]. If the level of expression was <50 for all the selected tissues, data are not shown.

Cell wall metabolism	Locus	Protein name	Expression level					
			Pollen grain		Pollen tube		Pistil	
			Dry	Imbibed	4 h *in vitro*	Semi *in vivo*	stigma	ovary
**Lyase**								
PL1	At1g04680	Probable pectate lyase 1	<50	<50	<50	<50	681.0	1,457.2
	At1g14420	Probable pectate lyase 3	3,922.2	3,514.5	3,591.2	1,171.3	<50	<50
	At2g02720	Probable pectate lyase 6	3,608.0	3,599.7	3,533.1	4,007.9	<50	<50
	At3g01270	Probable pectate lyase 7	6,694.0	6,572.3	6,546.1	7,161.1	166.1	112.7
	At4g24780	Probable pectate lyase 18	<50	<50	<50	<50	1,969.1	1,173.3
	At5g09280	Major pollen allergen like protein	<50	<50	<50	3,192.1	<50	<50
	At5g15110	Probable pectate lyase 18	3,865.4	3,782.2	3,390.4	625.1	<50	<50

PL. Pectin Lyase. 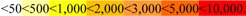

### 4.4. Glycoside Hydrolases

Glycoside hydrolases (GHs) are enzymes that catalyze the hydrolysis of glycosidic linkages between two or more glycosides or between a carbohydrate and a non-carbohydrate moiety. In 1991, GHs were classified into 35 families [192] and to date they are divided into over 100 families [193]. GHs involved in the reassembly of the pollen tube cell wall belong to the GH3, 9, 10, 17, 28, 35, 43 and 51 families. Their activities as well as their possible functions during pollen tube growth are presented in Table 5.

**Table 5:** Summary of the main glycoside hydrolase (GH) families possibly involved in the remodeling of the pollen tube cell wall.

Family	Known activities	Possible function during pollen tube growth
GH3	β-d-xylosidase (EC 3.2.1.37) α-l-arabinofuranosidase (EC 3.2.1.55) β-glucosidase (EC 3.2.1.21)	Degradation of xylan/arabinoxylan Degradation of RG-I, arabinogalactan proteins.
GH9	*Endo*-(1→4)-β-glucanase (EC 3.2.1.4) Cellobiohydrolase (EC 3.2.1.91) β-glucosidase (EC 3.2.1.21)	Degradation of cellulose and XyG backbone May control the diameter of the pollen tube. May help to digest the cell wall of the stigma to facilitate the pollen tube penetration in the female tissues.
GH10	*Endo*-(1→4)-β-xylanase (EC 3.2.1.8)	Degradation of xylan
GH17	*Endo*-Glucan (1→3)-β-glucosidase (EC 3.2.1.39) Glucan (1→3)-β-glucosidase (EC 3.2.1.58) *Endo*-(1→3-1→4)*-*β-glucanase (EC 3.2.1.73) β-(1→3)-glucanosyltransglycosylase (EC 2.4.1.)	Degradation of callose or β-mixed-(1→3, 1→4) glucans. May promote pollen germination and control the mechanical properties of the pollen tube cell wall during elongation.
GH28	Polygalacturonase (EC 3.2.1.15) *Exo*-polygalacturonase (EC 3.2.1.67) *Exo*-polygalacturonosidase (EC 3.2.1.82) Rhamnogalacturonase (EC 3.2.1.171) Rhamnogalacturonan α-l-rhamnopyranohydrolase (EC 3.2.1.40) *Endo*-xylogalacturonan hydrolase (EC 3.2.1.-)	Degradation of weakly esterified HG: cell wall loosening. May control the stiffness of the tube during elongation and/or help to digest the cell wall of the stigma thus facilitating the penetration of the tube. Degradation of RG-I backbone Degradation of xylogalacturonan
GH31	α-xylosidase (EC 3.2.1.177)	Degradation of XyG or RG-II side chains
GH35	β-galactosidase (EC 3.2.1.23) *Exo*-β-(1→4)-galactanase (EC 3.2.1.-)	Degradation of RG-I, XyG and/or arabinogalactan proteins. Turn over of pollen tube cell wall. Pollen tube guidance
GH43	β-d-xylosidase (EC 3.2.1.37) α-l-arabinofuranosidase (EC 3.2.1.55)	Degradation of xylan/arabinoxylan Degradation of RG-I, arabinogalactan proteins, arabinoxylan.
GH51	α-l-arabinofuranosidase (EC 3.2.1.55)	Degradation of RG-I, arabinogalactan proteins, arabinoxylan.

*Endo*-(1→4)-β-glucanases and *endo*-(1→3)-β-glucosidases are members of the GH9 and GH17 families, respectively (Table 5, Table 6). In *A. thaliana*, 25 genes are encoding GH9 proteins and 49 the GH17 proteins. Among the 25 putative *CELLULASES*, two are strongly expressed in pollen grains and three in *in vitro*-grown pollen tubes (Table 6). Four members of the GH17 family are expressed in *in vitro*-grown pollen tubes (Table 6). Three and five genes encoding the GH9 and GH17 are also found in the pistil tissues, respectively. Twenty years ago, these two enzyme activities were assayed in pistils and anthers of bean and they were linked to the cell wall disruption occurring during the release of the pollen grain from the anther and during the penetration of the pollen tube through the stigma [194]. Recently, and despite its low abundance in the pollen tube cell wall, it was shown that cellulose plays a crucial role by influencing the diameter of *in vitro*-grown pollen tubes [195]. As described previously, the cell wall of fast-growing pollen tubes is enriched in callose and several studies have shown that moderate β-(1→3)-glucanase treatments are able to stimulate the germination of pollen grains [53,196]. Moreover, it was reported that *exo*-β-glucanases might play an important role in the regulation of pollen tube elongation in lily [197]. On the contrary, treatments with inhibitors of glucosidases severely inhibited the growth of pollen tubes [198]. It was hypothesized that the mechanical properties of callose and more precisely the resistance to lateral deformation of the tube was regulated by glucanases [53] (Table 5).

**Table 6:** Analyses of the expression profile of glycoside hydrolase (*GHs*) genes in pollen grains, pollen tubes and in the pistil of *A. thaliana*. Data were collected from eFP Browser [148]. Proteins are named according to Magrane and the UniProt consortium [149] and to Hruba *et al*. [199] for the GH3 family. Pollen grain and pollen tube data are from Qin *et al*. [93] and the pistil data from Swanson *et al*. [151]. If the level of expression was <50 for all the selected tissues, data are not shown.

Cell wall metabolism	Locus	Protein name	Expression level					
			Pollen grain		Pollen tube		Pistil	
			Dry	Imbibed	4 h *in vitro*	Semi *in vivo*	stigma	ovary
**Glycoside Hydrolases**								
GH3	At1g02640	β-d-xylosidase	<50	<50	<50	<50	66.1	227.3
	At1g78060	β-d-xylosidase	<50	<50	<50	<50	149.2	401.8
	At3g19620	β-d-xylosidase	<50	<50	<50	<50	388.6	76.9
	At3g47000	β-d-xylosidase	<50	<50	<50	<50	190.2	330.6
	At3g62710	β-d-xylosidase	4,780.4	4,342.3	4,813.6	6,223.6	73	51.7
	At5g09730	β-d-xylosidase 3/α-l-Arabinofuranosidase	<50	<50	<50	<50	803.5	2,807.8
	At5g10560	β-d-xylosidase	<50	<50	<50	<50	191.5	508.8
	At5g20940	β-d-xylosidase	<50	<50	<50	<50	<50	62.9
	At5g20950	β-d-xylosidase	<50	<50	<50	<50	741.4	1,468.4
	At5g49360	β-d-xylosidase 1/α-l-Arabinofuranosidase	<50	<50	<50	<50	193.9	921.4
	At5g64570	β-d-xylosidase	<50	<50	<50	<50	324.3	1,586.8
GH9	At1g70710	Endo-(1→4)-β-glucanase (CEL1)	<50	<50	<50	<50	732.8	1,921.9
	At1g71380	Endo-(1→4)-β-glucanase (CEL3)	<50	71.0	665.4	2,823.2	<50	57.8
	At2g44560	Endo-(1→4)-β-glucanase	2,345.4	2,152.9	2,492.6	1,224.1	<50	<50
	At3g43860	Endo-(1→4)-β-glucanase	5,932.2	5,925.4	5,738.7	3,735.4	<50	<50
GH9	At4g02290	Endo-(1→4)-β-glucanase (CEL4)	<50	<50	<50	<50	<50	1,223.5
	At5g49720	Endo-(1→4)-β-glucanase (RSW2/KOR1)	<50	<50	<50	<50	822.2	1,027.5
GH10	At4g33850	GH 10 protein	1,571.9	1,369.8	1,495.2	205.7	<50	<50
	At4g33860	Endo-(1→4)-β xylanase, putative	1,571.9	1,369.8	1,495.2	205.7	<50	<50
GH17	At2g05790	Putative β-(1→3)-glucanase	<50	<50	<50	<50	1,054.1	2,723.2
	At3g07320	Putative β-(1→3)-glucanase	<50	<50	<50	<50	482.9	1,241.9
	At3g55430	Putative β-(1→3)-glucanase	3,815.6	3,499.7	3,184.8	1,146.9	504.2	824.6
GH17	At4g26830	Putative β-(1→3)-glucanase	<50	<50	221.1	3,284.7	<50	<50
	At5g20390	Putative β-(1→3)-glucanase	3,190.6	3,251.1	3,427.8	365	<50	<50
	At5g42100	Glucan endo-(1→3)-β-glucosidase 10	<50	<50	<50	<50	726.5	1,402.3
	At5g55180	β-(1→3)-glucanase like	<50	<50	<50	<50	258.2	1,247.9
	At5g64790	β-(1→3)-glucanase	2,007.8	1,991.2	1,972.5	1,439.2	<50	<50
GH28	At1g02790	Exopolygalacturonase (PGA4)	6,048.4	5,842.1	5,885.1	5,376.4	137.3	82.5
	At2g23900	Putative polygalacturonase	3,341.6	3,090.6	3,228.7	2,308.4	<50	<50
	At3g07820	Polygalacturonase (PGA3)	6,791.1	7,077.4	6,983.5	7,731.2	137.5	63.1
	At3g07850	Exopolygalacturonase	7,694.0	7,415.6	7,187.2	3,814.6	<50	<50
	At3g14040	Exopolygalacturonase	7,694.0	7,415.6	7,187.2	3,814.6	<50	<50
	At4g23820	Putative polygalacturonase	<50	<50	<50	<50	726.1	1,214.5
	At5g48140	Putative polygalacturonase	5,423.3	5,327.8	5,417.7	5,266.6	<50	<50
GH31	At1g68560	α-xylosidase (XYL1)	<50	<50	<50	<50	500.4	765.4
	At3g45940	α-xylosidase (XYL2)	<50	<50	<50	<50	101.3	171.9
GH35	At2g16730	β-galactosidase (BGAL13)	3,753.9	3,752.6	3,219.2	550.5	<50	<50
	At2g28470	β-galactosidase (BGAL8)	<50	<50	<50	<50	2,437.4	2,075.1
	At4g35010	β-galactosidase (BGAL11)	4,248.3	4,056.9	4,264.4	5,783.1	77.7	<50
	At4g36360	β-galactosidase (BGAL3)	<50	<50	<50	<50	598.5	1,462.5
GH43	At3g49880	β-xylosidase	<50	<50	<50	<50	77.8	134.7
GH51	At3g10740	α-l-Arabinofuranosidase	<50	<50	<50	<50	184.9	402.8

GH. Glycoside Hydrolases, CEL. Cellulase, RSW.Radially Swollen, KOR. Korrigan, PGA. Polygalacturonase, BGAL. β-galactosidase, XYL. xylosidase. 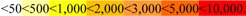

Polygalacturonases (PGs) are involved in the HG degradation. They were characterized in the pollen from corn and other Poales [200] and have since been identified in various plants including rice, pea, tomato and arabidopsis [143,190,201,202]. PGs belong to the GH28 family composed of 69 genes in *A. thaliana* [203,204]. In 2000, Torki *et al*. [205] showed that 7 *PG* genes were strongly expressed in *A. thaliana* flowers. Publicly available microarray data are consistent with this result as 6 *PG* genes are strongly expressed in pollen tubes and one is expressed in the pistil (Table 6). Together with the strong expression of more than 10 *PMEs* and 4 *PLLs* in pollen tubes, these data suggest an important remodeling of the HG during pollen tube growth. PGs are involved, during pollen maturation of *Brassica campestris*, in the intine and/or exine formations [206,207] and in *Turnera subulata*, PGs are implicated in self-incompatibility [208]. PGs are released upon rehydration of triticale pollen grains [146] and are also detected in the tip region of pollen tubes in *Brassica napus* during papillar cell penetration [209] suggesting that PGs are probably involved in the loosening of the stigma cell wall during pollination. As PGs degrade weakly methylesterified HG and that the demethylesterification of methylesterified HG by PMEs at the tip of the tube is accompanied by the release of protons, it was suggested that this local change of pH in the pollen tube cell wall may promote PG activity [58,210]. This activation of PGs might then control the loosening of the pollen tube cell wall back from the tip and then promote the proper pulse growth of the tube (Table 5). Alternatively, pollen tube PGs may also affect the loosening of the stigma and transmitting tract cell wall to facilitate the penetration of the pollen tube (Table 5).

β-galactosidases (BGALs) belong to the GH35 family. BGALs can act on different substrates including arabinogalactan proteins, galactolipids, RG-I and RG-II side chains of pectin and XyG releasing galactose [211]. Eighteen genes are encoding BGALs in the *A. thaliana* genome and the functional genomics analysis reveals that *BGAL* expression levels are high in mature leaf, root, flower and silique but are low in young seedling [212]. Publicly available microarray data (Table 6) show that 2 *BGALs* are highly expressed in mature pollen grains and 2 others are highly expressed in the pistil confirming the results obtained by Ahn *et al*. [212]. Other *BGAL* genes are also strongly expressed during the microspore mitosis in developing pollen grains [199]. In rice, 35 genes are encoding BGALs; among them, two are strongly expressed in dry pollen grains [213] (Table 6). As mutants defective in BGAL have impaired fertility, it was hypothesized that BGAL may be involved in the pollen tube wall turnover in *Brassica campestris* by hydrolyzing arabinogalactan [214]. In 1995, it was suggested in tobacco that BGALs could be secreted from the pollen tube and released in the stylar transmitting tract to modify the branching pattern of arabinogalactan involved in the guidance of pollen tubes [215,216]. In tobacco, *BGAL* mRNAs are accumulated during the formation of pollen grains, presumably stored for future use during pollen germination and pollen tube growth as BGAL activities were detected in growing pollen tubes [217]. In rice, 6 isoforms of putative BGALs were detected by proteomic [143]. Finally, it was also suggested that BGALs might also be required for PME activity [218,219]. Transcriptomic data showed very strong pollen specific expression profile of one β-xylosidase gene in mature dry pollen grains [199], and in *in vitro* and semi-*in vivo* grown pollen tubes. The exact substrate of this enzyme is not known but it may degrade several cell wall polymers such as xylan, xylogalacturonan and possibly *N*-linked glycoproteins. All the other putative xylosidases (12 genes that belong to the families GH3 and GH31) are only expressed in the pistil (Table 6). In addition, transcriptomic data do not show a strong expression of arabinofuranosidase genes in the pollen (Table 6). This is somehow surprising considering that Ara is one of the main carbohydrates present in the cell wall of *A. thaliana* pollen tubes [25]. It may suggest that the Ara containing polymers are not remodeled and/or that arabinofuranosidases originate from the female tissues (Table 6). The pattern of expression of α-xylosidase genes (GH31) is close to the one found for the arabinofuranosidase genes. α-xylosidase genes are not strongly expressed in dry pollen grains or pollen tubes. However, two α-xylosidase genes are expressed in the pistil (Table 6). Analyses of the *A. thaliana Atxyl1* mutants show reduced α-xylosidase activity, altered XyG composition and shorter siliques compared to the wild type. Moreover *pAtxyl1::GUS* expression is strong in the style [220]. It suggests that AtXYL1 may remodel the XyG of pollen tubes. However, it is difficult to directly implicate this α-xylosidase activity in pollen tube growth as seed production was apparently not affected.

By comparing the proteome of rice pollen grains and pollen tubes, Dai *et al*. [191] showed that several proteins involved in the cell wall biosynthesis and remodeling are either up- or down-regulated. Several RGP, PME, PG and EXPB isoforms are up-regulated upon pollen germination whereas others PGs, xylanases and EXPBs are down-regulated indicating that several proteins are dedicated to pollen germination and others to pollen tube growth. The authors suggested that the down-regulated proteins in the pollen tube may result from their release into the culture medium. In the *in vivo* context, the release of these proteins in the pistil tissues may facilitate the pollen tube growth. Finally, glycosylated and/or phosphorylated proteins were detected and contributed to the generation of new isoforms [191]. The activity of enzymes is commonly regulated by phosphorylation, glycosylation and/or interaction with specific inhibitors but more studies are required to assess the fine tuning and the exact function of these posttranslational modifications in pollen tube growth [191].

## 5. Mechanical Properties of the Cell Wall Network during Pollen Tube Growth

In recent years, A. Geitmann’s lab has studied the mechanical properties of pollen tubes using micro-indentation and finite element technique [63]. In *A. thaliana*, lily and *S. chacoense*, it is observed that the tip region is elastic and the shank of the tube is rigid [26,53]. The modification of the cell wall mechanical properties using exogenous and moderate pectinase concentrations promotes pollen tube growth and the overall stiffness of the pollen tube decreased in both the apical and distal regions [53] suggesting that pectins are important components in respect to the cell wall mechanics [63]. As expected, an increase of the pectinase concentrations results at first by in apical swelling and ultimately bursting of the pollen tube. By contrast, PME treatments of pollen tubes increase the cellular stiffness at the apex and reduce the visco-elasticity. It reveals that the gradient from methylesterified (in the hemisphere-shape tip) to de-methylesterified (in the cylindrical shank) HG increases the cell wall rigidity [30] by promoting calcium cross-links with the negatively charged carboxylic groups of HG. This was also observed by Rounds *et al*. [45] using PI on pollen tubes treated with PME. A dramatic increase of the PI fluorescence at the apex of treated pollen tubes was detected due to an increase of demethylesterified HG. The authors suggested that PI interacted with demethylesterified HG by competing with calcium.

Despite its low abundance, cellulose plays an important role in stabilizing the pollen tube tip wall especially in the transition zone between the tip and the shank [13]. Cellulose microfibrils display an oblique orientation along the pollen tube wall in *Pinus* [27] whereas a more longitudinal orientation is observed in *Lilium*, *S. chacoense* and *A. thaliana* suggesting that cellulose microfibrils are not the main stress bearing component against turgor pressure [26,195,221]. However, cellulose is of main importance in the mechanical stabilization of the tip region. Cellulase treatments or inhibition of cellulose crystal formation with drugs resulted in larger pollen tube diameters and promoted tip swelling and eventually bursting [195] as observed with the *cellulose synthase* mutants. Similar phenotypes were observed on *Petunia* and lily pollen tubes treated with 2,6-dichlorobenzonitrile (DCB), a cellulose biosynthesis inhibitor [222] or on conifer pollen tubes treated with isoxaben [56]. Recently, Derksen *et al*. [54] have shown that the cell wall structure of tobacco pollen tubes is organized both at the tip and back from the tip of 40–50 nm spaced lattice of continuous fibers that will allow intake of substantial size molecules from the surroundings.

*In planta*, XyGs are known to interact by hydrogen bounding with cellulose microfibrils but no information is available in pollen tubes. *O*-acetyl groups and the fucose residues of XyG do not seem to play a major role in the interaction with cellulose. *In vitro* experiments have shown that acetylated or de-acetylated XyG can cross-link similarly cellulose microfibrils [223]. In contrast, the presence of galactosyl residues appeared to be important in promoting the interaction with cellulose. XyG lacking Gal residues self-associated and did not interacted with cellulose [223]. Moreover, *A. thaliana* mutants with XyG lacking xylosyl and galactosyl residues showed a reduction of the tensile strength [224] and abnormal bulging in root hairs, possibly due to impaired cellulose-XyG assembly [225,226]. All together, these data suggests that the high levels of fucosylation and *O*-acetylation in the *A. thaliana* pollen tube XyG and perhaps in other species may prevent a strong interaction with cellulose microfibrils at the tip, promoting the fast growth of the pollen tube. Moreover, the high level of *O*-acetylation in XyG pollen tubes may also modulate the interaction between cell wall polymers and/or hinder enzymatic degradation [227] perhaps from enzymes originating from the pistil. Very recently, in root hairs of *A. thaliana*, a new branching pattern of XyG was characterized with GalA residues instead of Gal [228]. This motif was not detected in *A. thaliana* pollen tubes but it indicates clearly that tip-polarized cells can have specific XyG compositions to modulate the interaction with cellulose microfibrils and promote fast growth.

The amorphous callose is also important in the mechanical properties of the pollen tube cell wall. Treatments of pollen tubes with lyticase, an enzyme able to degrade callose, increase the diameter of the pollen tube, reduce the cellular stiffness and increase the cellular viscoelasticity in the distal part of *S. chacoense* pollen tubes suggesting that the callose wall may function in resistance to compression and/or tension stresses [53]. Using a combination of PME, pectinase and lyticase on fixed pollen tubes, Chebli *et al*. [26] suggested that cellulose and pectin are closely linked in the tip region of *A. thaliana* pollen tubes as demonstrated in the *A. thaliana* primary cell wall [229] and in the shank of the tube, a tight network of cellulose and callose is formed. Recently, using cellular force microscopy on lily pollen tubes, Vogler *et al*. [230] revealed also that the apparent stiffness of the pollen tube was lower at the tip than in the shank. However, they suggested that these differences are not originating from the distribution of the cell wall polymers or the thickness of the cell wall but solely due to the geometry of the pollen tube.

## 6. Conclusions

**Figure 4:**
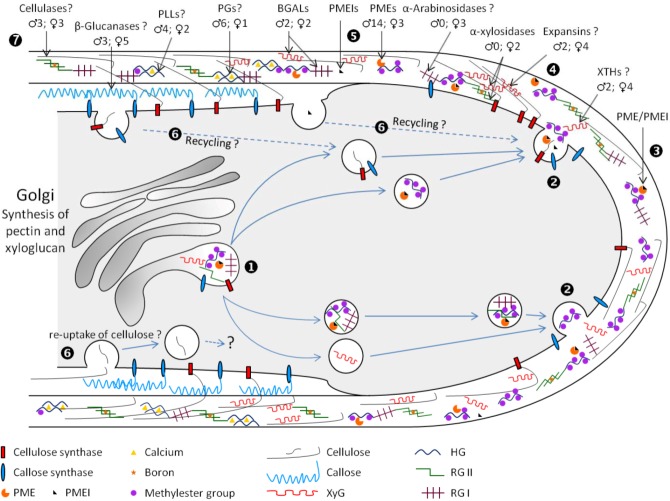
Model presenting the deposition of the main polysaccharides composing the pollen tube cell wall and its possible remodeling with proteins originating from the pollen tube or the pistil. (➊) Callose and cellulose synthases as well as XyG and pectin (HG, RG-I and RG-II) are carried within Golgi-derived vesicles. HG is synthesized in a highly methylesterified form and may or not be transported together with PME/PMEI complexes. (➋) The vesicles are directed to the plasma membrane in the sub-apical zone of the pollen tube tip where they fuse and release their contents (polysaccharides and/or remodeling proteins) in the cell wall. XyG is released under its final fucosylated form. Callose and cellulose synthases stay embedded in the membrane whereas PME/PMEI complexes and the other cell wall remodeling enzymes are released in the apoplast (➌). (➍) XyG and cellulose microfibrils probably interact loosely in the pollen tube tip and/or XTHs and expansins may facilitate the loosening process. It is not known if the RG-II borate dimer is synthesized in the Golgi and secreted under its final form or formed in the cell wall with exogenous boron coming from the culture medium or the pistil. (➎) PMEs are activated after their separations from PMEIs. PMEs are demethylesterifying the HG that promote the fixation of calcium ions between several parallel HG chains, thus reinforcing the cell wall rigidity. PMEs are hypothesized to remain in the apoplast in the shank of the pollen tube. PMEIs may be degraded by proteases or recycled by endocytosis (➏). Similarly, it is hypothesized that the excess of cellulose and callose synthases may eventually be recycled by endocytosis. Similarly, cellulose microfibrils may also be endocytosed suggesting the action of cellulases. (➐) Comparable process might be observed with the degradation of callose with β-glucanases. The main class of enzymes (cellulases, β-glucanases, PLLs, PGs, PMEs, BGALs, α-xylosidases and α-arabinosidases, expansins and XTHs) possibly implicated in the remodeling and/or degradation of the pollen tube cell wall are presented. Numbers below the proteins correspond to the number of genes highly expressed in the pistil (♀) and the pollen tube (♂) of *A. thaliana*. BGALs, β-galactosidases; PGs, polygalacturonases; PLLs, pectate lyases-like; PMEs, pectin methylesterases; PMEIs, pectin methylesterase inhibitors; RG-I, rhamnogalacturonan-I; RG-II, rhamnogalacturonan-II; XTHs, xyloglucan *endo*-transglucosylase hydrolases; XyG, xyloglucan. Objects are not drawn to scale.

Over the last 25 years, the use of *in vitro* systems has been very valuable and a lot of information was collected concerning the distribution of the cell wall polymers in many species, but mostly focused on HG, callose, cellulose and arabinogalactan-proteins. In *A. thaliana*, functional genomics approaches have allowed the characterization of several genes implicated in the biosynthesis of pollen tube cell wall polysaccharides (mostly callose and cellulose). However, considering the large number of genes necessary to synthesize pectin, very few of them have been functionally characterized (several in the RG-II biosynthesis) and so far, none involved in the synthesis of XyG. Similarly, very few pollen genes involved in the remodeling of the pollen tube cell wall have been experimentally characterized. In addition, in the *in vitro* studies of pollen mutants, it is sometimes difficult to compare the data due to the use of different culture media and conditions, which may enhance or reduce the observed phenotypes. In many cases, a mutation completely inhibits pollen germination thus preventing the investigation of functional studies during pollen tube growth. Moreover, very little information is available on “non model” pollen tube species especially in gymnosperms and monocots. However, in recent years, the number of sequenced genomes from evolutionary divergent species has increased and will probably provide important information in the search of orthologous genes as it was done with *CALLOSE SYNTHASE5*. The development of new probes targeting the cell wall components and the improvement of the technology have also allowed more detailed studies on the cell wall biochemistry and the mechanic of the pollen tube cell wall. Finally, to increase the complexity of the system during their journey pollen tubes travel in the female tissues where additional cell wall interactions, cross-linking, modification, degradation and recycling can occur (Figure 4). The challenge will be to integrate all the parameters from protein signaling and trafficking to cell wall deposition and remodeling in the *in vivo* context in response to extracellular cues. Solving this complex network of information will unravel the secret of pollen tube tip expansion and guidance.
